# (Electro)chemical and Mechanical Degradation Studies of Commercial Proton Exchange Membranes in HCl/Cl_2_ Systems for Hydrogen/Chlorine Production

**DOI:** 10.1002/open.202500549

**Published:** 2026-05-10

**Authors:** Alen Rupnik, Janvit Teržan, Gleb Veryasov, Miha Grilc, Blaž Likozar

**Affiliations:** ^1^ Department of Catalysis and Chemical Reaction Engineering National Institute of Chemistry Ljubljana Slovenia; ^2^ Materials graduate study program University of Nova Gorica Nova Gorica Slovenia; ^3^ Laboratory for demonstration of H_2_ and CO_2_ technologies National Institute of Chemistry Kisovec Slovenia; ^4^ TotalEnergies One Tech Belgium Feluy Belgium

**Keywords:** fluoropolymer degradation, hydrogen production, PEM electrolysis, PEM membrane degradation studies, PFSA polymers

## Abstract

Proton exchange membrane (PEM) electrolysis is a cornerstone technology for sustainable hydrogen production. In addition to water splitting, it is also used in hydrochloric acid (HCl) electrolysis to convert industrial HCl waste streams into valuable chlorine and hydrogen. However, such highly corrosive environments present unique material challenges. This study evaluates the mechanical, thermal, and chemical durability of various commercially available perfluorosulfonic acid (PFAA) membranes—including several Nafion and Aquivion variants—under harsh HCl/Cl_2_ electrolysis conditions. Characterization techniques included contact angle measurements, tensile strength testing, thickness analysis, scanning electron microscopy (SEM), Fourier‐transform infrared spectroscopy (FTIR‐ATR), and long‐term electrolysis testing. While Nafion membranes demonstrated good mechanical strength, they showed notable surface degradation and chemical instability under acidic conditions. In contrast, Aquivion E98‐09S exhibited superior chemical resilience and structural integrity, even after 300 h of continuous operation in 20 wt% HCl. These findings underscore the importance of tailored membrane selection for chlorine‐rich electrochemical systems and offer valuable insights for the development of more robust and durable PEM materials for halogen‐based electrolysis technologies.

## Graphical abstract

Durability of proton exchange membranes (PEMs) in hydrochloric acid/chlorine (HCl/Cl_2_) electrolysis is assessed. Mechanical, thermal, and chemical properties of commercial Nafion and Aquivion PEMs are compared using microscopy, spectroscopy, and long‐term tests. Aquivion E98‐09S shows superior stability, highlighting material choices for robust halogen‐based electrolysis.

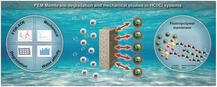

## 1 Introduction

Proton exchange membrane (PEM) electrolysis has proven to be a promising technology for clean and efficient hydrogen production and plays a pivotal role in the transition toward a sustainable energy economy [1]. With its ability to convert renewable electricity into hydrogen through water electrolysis, PEM electrolysis has the potential to address critical challenges in energy storage, transport, and industrial processes [2, 3]. Crucial to the performance and durability of PEM electrolysis systems is the integrity of the PEM itself, a critical component that facilitates proton transport while separating reactant gases. The PEM membrane, which usually consists of a polymer electrolyte impregnated with proton‐conducting materials, is an important key interface within the electrolysis cell [4]. During the electrolytic process, the PEM membrane is exposed to various harsh conditions, including high temperatures, exposure to reactive species, and mechanical stresses. Together these factors collectively contribute to the gradual degradation of the membrane and affect its ionic conductivity, mechanical integrity, and long‐term stability. Consequently, the study of PEM membrane degradation and its impact on electrolysis performance is an important research endeavor, especially in highly corrosive environments [5].

The significance of this study goes beyond the realm of fundamental research; it has direct implications for the wider adoption of PEM electrolysis technology in real‐world applications. As global demand for clean hydrogen increases to meet ambitious decarbonization targets, effective hydrogen production is a necessary future requirement, for a carbon‐free energy source and for successfully achieving a net‐zero future as proposed by the European Union [6]. There are various options for hydrogen production, such as methane steam reforming (MSR), the water gas shift reaction (WGS), and PEM electrolysis (PEMEC) using different electrolytes. There is a growing interest for alternative electrolytes to pure water, such as chlor‐alkali [7] electrolytes, which serve as a source of chlorine and hydrogen. Recently, there has been a resurgence of interest in halogens within the realm of chemistry and electrochemistry. These elements have attracted a lot of attention due to their exceptional reactivity and diverse applications [8, 9]. For example, hydrochloric acid (HCl), which is a byproduct in the production of polycarbonate or polyurethane, could be used as a feedstock [10]. In this case, the on‐site conversion of HCl into hydrogen and chlorine would be of great economic and safety benefit. Since HCl is a strong acid and thus very corrosive, design of a stable‐operating cell for electrocatalytic decomposition of the latter would demand careful selection of construction materials. While structural components of electrolyzers can be fabricated from HCl‐resistant materials such as PTFE, the selection of appropriate proton exchange membranes remains a major challenge due to the need to combine proton conductivity with long‐term chemical and mechanical stability in highly corrosive acidic and chlorinated environments [11].

This scientific paper aims to highlight the critical importance of investigating the degradation of PEM in the context of PEM electrolysis for chlorine‐containing acidic systems. By exploring the mechanical properties and chemical stability against acidic and chlorine‐containing media in conjunction with thermal stability and electrochemical stability studies, researchers and industry process engineers can use this data as a tool for quick screening for suitable commercially available PEM membranes for HCl/Cl_2_ electrolysis systems and similar halogen chemistry. Understanding these intricate relationships not only aid in the development of more resilient and durable PEM materials but also suggests strategies to optimize system performance and mitigate performance losses associated with membrane deterioration. We can find individual studies of specific membranes properties [12] and studies on membranes for unique applications, such as steam or other gas permeation studies [13, 14], but more comprehensive research about many of commercial membranes is still lacking.

Perfluorosulfonic acid (PFSA) polymers, such as Nafion and Aquivion, are still the most commonly used materials for proton exchange membranes in polymer electrolyte membrane cells, mainly due to their high proton conductivity, chemical stability, and mechanical robustness. These membranes are structurally composed of a hydrophobic polytetrafluoroethylene (PTFE) backbone with perfluorinated side chains that end in sulfonic acid groups, which phase‐separate into hydrophilic and hydrophobic domains upon hydration, forming well‐connected aqueous channels that facilitate proton conduction while maintaining mechanical integrity [15]. The conduction mechanism predominantly follows the Grotthuss and vehicular pathways, in which protons are transported either by hopping between hydrogen‐bonded water molecules or by moving alongside water clusters, respectively. They are ideally suited to harsh conditions, making them key components in advanced energy technologies and prompting ongoing research for further improvements. Nevertheless, the stability of proton exchange membranes plays a crucial role in the performance and longevity of electrolysis cells, especially in hydrogen production. Nafion, the industry standard, exhibits high proton conductivity and mechanical robustness, but suffers from degradation due to chemical, mechanical, and thermal stress [16]. One of the major challenges in water electrolysis is the interaction with reactive oxygen species (ROS), which leads to the loss of sulfonic acid groups, membrane thinning, and eventual failure [16]. To combat these issues, recent studies have explored various strategies for improving membrane stability. Hybrid membranes, such as those containing quaternized polyphenylene oxide (qPPO) and polyvinyl alcohol (PVA), have demonstrated increased ionic conductivity (up to 151 mS/cm) and enhanced mechanical strength, significantly outperforming conventional Nafion membranes [17]. Additionally, improved thermal conductivity has been investigated for its capacity to mitigate localized heat accumulation, which accelerates degradation [18]. Recent research aims to address these challenges by developing composite membranes that incorporate inorganic fillers—such as sulfonated zirconium‐based metal‐organic frameworks and sulfonated graphene oxide—to enhance water retention and proton conductivity under low‐humidity conditions [19, 20]. In parallel, chemical modifications are being explored to improve oxidative stability and mechanical durability.

At the same time, increasing attention is being given to environmentally benign alternatives, such as nonfluorinated or partially fluorinated PEMs, which can reduce the environmental footprint of conventional PFSA membranes [21]. These innovations continue to drive the development of PEM materials toward more durable, cost‐effective, and sustainable fuel cell/electrolyzer technologies.

Similarly, to water electrolysis, HCl electrolysis also faces these challenges, as both hydrochloric acid and chlorine are highly corrosive and reactive species, meaning chlorine can react with sulfonic acid groups in perfluoro‐sulfonic membranes, leading to chemical degradation of the membrane. The reaction occurs primarily through oxidative attack of chlorine and chlorine‐derived reactive species, such as HOCl and OCl^−^ ions [22, 23, 24]. Additionally, membrane conductivity is influenced not only by chemical stability but also by thermal management. Enhancing thermal conductivity has been explored as a strategy to reduce local heat accumulation, which can accelerate degradation and affect long‐term performance [18].

The impact of such stability improvement on the conductivity of electrolytic cells is substantial. While maintaining high proton transport efficiency is essential, excessive cross‐linking or filler addition can hinder ion mobility, creating a tradeoff between mechanical durability and conductivity. Overall, advancing PEM technology requires a balance between chemical resilience, thermal management, and optimized ion pathways to ensure stable and efficient electrolysis for long‐term hydrogen production.

## 2 Experimental

The study utilized a selection of several perfluoro sulfonic polymer membranes, including Nafion variants (HP, XL, 117, 211, 1110, N424, and N438) and Aquivion types (E98‐05, E98‐05S, and E98‐09S). The methods described in the following subsections were conducted on bespoke membranes using tailored methods and equipment specific for this research.

### 2.1 Mechanical and Structural Properties of Membranes

Different membranes have different mechanical properties that make them suitable for specific applications. Some membranes tear easily, while others, although more elastic, may warp under stress. Additionally, membrane thickness significantly influences cell conductivity and gas crossover, directly impacting performance and durability. Therefore, understanding these properties is crucial for end users when selecting the most suitable membrane for their needs.

#### 2.1.1 Wetting Angle

Contact angle measurements were performed using the Theta T200 optical contact angle goniometer (Biolin Scientific), which determines the angle by analyzing the shape of a water droplet placed on the membrane surface using high‐resolution side‐view imaging and digital curve fitting. Membrane strips were securely fixed to the sample table to ensure a stable measurement surface. A drop of deionized water was carefully deposited onto the membrane surface, and the contact angle was recorded using the instrument's built‐in high‐resolution camera. The measurement was analyzed using specialized processing software, which accurately determined the wetting angle based on the drop shape and surface interactions.

#### 2.1.2 Membrane Thickness

As previously mentioned, membrane thickness significantly impacts conductivity and can either increase or reduce gas transfer. To assess this, we compared the thickness specified by the manufacturer with our own measurements using a high‐precision digital micrometer from Mitutoyo. Each membrane strip was measured at 10 randomly selected points, and the average thickness was compared to the specified value to evaluate consistency and accuracy.

#### 2.1.3 Tensile Strength

Tensile strength of the membranes was measured using a Zwick Roell Z010 universal testing machine. Membrane strips, sized 8 cm × 1 cm (L × W), were securely fixed onto the holder for testing. The average thickness value was then used for tensile strength calculations, as detailed in the previous chapter.

### 2.2 Thermal Degradation Study in Hydrochloric Acid

The testing campaign was carried out with a batch setup, comprising of two electrodes and a holder for PEM membranes in between. The setup was submerged in a 20 wt% HCl solution. The stability tests were conducted at 60°C for 48 h in order to determine the initial membrane resistance to hydrochloric acid at elevated temperatures.

A custom membrane holder was fabricated by the help of a fused deposition modeling (FDM) 3D printer. The material used was PET‐G since it is easily printed and exhibits acceptable resilience when exposed to a 20 wt% hydrochloric acid solution.

#### 2.2.1 Scanning Electron Microscopy

The degradation was initially evaluated by scanning electron microscopy (SEM). The images of virgin membranes were compared to the used membranes. Before the SEM measurement, the membranes were cleaned with distilled water and air dried before analysis. We used the Carl Zeiss SEM microscope (HR‐SEM Zeiss Supra 35 VP). In order to be able to perform SEM analysis, all the samples were spray coated with a ∼ 6‐nm layer platinum.

#### 2.2.2 FTIR‐ATR

Fourier‐transform infrared spectroscopy in attenuated total reflectance mode (FTIR‐ATR) was used to analyze the chemical structure and potential degradation of the proton exchange membranes before and after exposure to hydrochloric acid electrolysis conditions. This technique was used to assess changes in functional groups, particularly sulfonic acid (‐SO_3_H) groups, and to identify potential oxidative degradation pathways.

FTIR‐ATR spectra were recorded using a PerkinElmer Spectrum 3 with a diamond GladiATR attachment from PIKE Technologies. The spectra were collected in the mid‐infrared region (4000–450 cm^−1^). Background correction was performed before each set of measurements to minimize interference from atmospheric CO_2_ and H_2_O.

The PEM samples were cut into approximately 1 cm × 1 cm sections and thoroughly rinsed with deionized water to remove residual electrolyte or contaminants. Before measurement, the samples were dried in a vacuum oven at 60 °C for 12 h to ensure that excess moisture that could interfere with spectral analysis was removed. Spectral comparisons of pristine, aged, and degraded membranes were used to identify chemical degradation mechanisms through changes in peak intensity and shifts in functional group.

### 2.3 Electrochemical Degradation Study of Aquivion E98‐09S Membrane

The chemical degradation of the Aquivion E98‐09S membrane was evaluated by integrating it into a MicroFlow electrolysis cell (Electrocell) equipped with a mixed metal oxide (MMO) anode and a platinum cathode, operating in 20 wt% HCl as the electrolyte. The electrolysis cell was run for 300 h at 400 mA constant current in potential ranges of up to 2.8 V, to determine long‐term stability of the membrane. Both the anolyte and the catholyte were continuously recirculated, while the chlorine was stripped with nitrogen and subsequently absorbed in 5 wt% aqueous NaOH scrubbing solution. Hydrogen was purged in order to prevent the pressure buildup in the cell. This setup allowed for the controlled evaluation of membrane degradation under realistic electrolysis conditions. After the study, the membrane was removed and the FTIR‐ATR was measured. The spectra were compared to those of the virgin membrane in order to detect any changes in functional sulfuric groups.

## 3 Results and Discussion

### 3.1 Mechanical and Structural Properties of Membranes

#### 3.1.1 Wetting Angle

**FIGURE 1:**
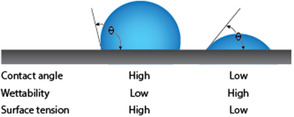
Contact angle comparison and properties for high vs. low contact angle.

Tested membranes have a different affinity for water and therefore a different surface tension. The water affinity is determined by measuring the wetting angle, where a higher angle means a lower wettability of the membrane and high surface tension [25]. In contrast, a lower value means a higher wettability and therefore a higher surface tension, as shown in Figure 1.

**FIGURE 2:**
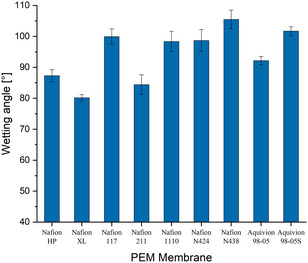
Wetting angle of different PEM membranes with standard deviation of measurements.

Among the tested membranes, Nafion XL and Nafion 211 exhibited the lowest wetting angles, indicating the highest surface wettability, while Nafion N438 showed the highest wetting angle and thus the most hydrophobic character. The overall results are shown in Figure 2.

**FIGURE 3:**
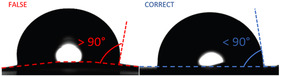
Illustration of contact angle measurement error due to membrane warping. Left: Incorrect measurement on a curved membrane surface resulting in a falsely high contact angle (>90°). Right: Correct measurement on a flat surface showing true wetting behavior (<90°).

We found that some membranes are highly hygroscopic and tend to warp—i.e., bend or curl—upon contact with water (Figure 3). This deformation affects the flatness of the surface, potentially leading to artificially high contact angle measurements. To ensure accuracy and reproducibility, membranes were affixed to a flat surface during measurement. This deformation may also be attributed to the membranes being in a dehydrated state prior to testing.

The measured contact angles show that membrane surface hydrophilicity varies significantly across different commercial PFSA membranes. Nafion 211 and XL, for example, exhibited the lowest contact angles, consistent with literature, indicating high surface wettability and water uptake capacity [12]. These membranes typically have a higher ion exchange capacity (IEC), which contributes to a denser hydrophilic domain network.

On the other hand, reinforced membranes such as Nafion N438 displayed much higher contact angles, indicating a more hydrophobic surface, possibly due to the influence of embedded PTFE support structures which reduce the exposed sulfonic group density at the surface. These findings align with previous observations that membrane reinforcement can compromise water affinity, potentially impacting proton transport under low‐humidity conditions [16].

Hydrophilicity is a key factor influencing water retention and ionic conductivity in PEM electrolysis, especially under dehydrating or high‐temperature conditions. Therefore, contact angle measurements can serve as a useful screening tool in the evaluation of membranes for harsh acidic electrolysis environments such as HCl/Cl_2_ systems.

#### 3.1.2 Membrane Thickness

The thickness of each membrane was measured on multiple points; the average values are collected in Table 1, together with the thickness value defined by the producer.

**TABLE 1:** Membrane thickness measured average vs. value specified by the producer.

Membrane	Average thickness, µm	Producer specifications, µm	Standard deviation, µm
Nafion 211	25	25	0,71
Nafion XL	25	25	0,45
Nafion HP	25	25	0,84
Nafion N424	370	330	5,40
Nafion N484	358	/	15,61
Nafion 1100	261	254	2,93
Nafion 117	188	183	1,58
Aquivion E98‐05	62	50	1,10
Aquivion E98‐05S	65	50	1,52
Aquivion E98‐09S	89	90	1,41

The membrane thickness for N424 differed by 40 µm. This could also be due to the micrometer‐wide surface tip, so we measured the thickness of the membrane across the uneven surface. On the other hand, the manufacturer may have specified the thickness of a single strand. However, this still emphasizes the uneven surface of such membranes, which is strongly influenced by the manufacturing process [26, 27]. Although surface roughness may contribute to variations in thickness measurements, optical imaging of the membrane surface alone cannot accurately reveal such unevenness. Cross‐sectional analysis would be required to fully assess the internal structure and layer differences.

#### 3.1.3 Tensile Strength

**FIGURE 4:**
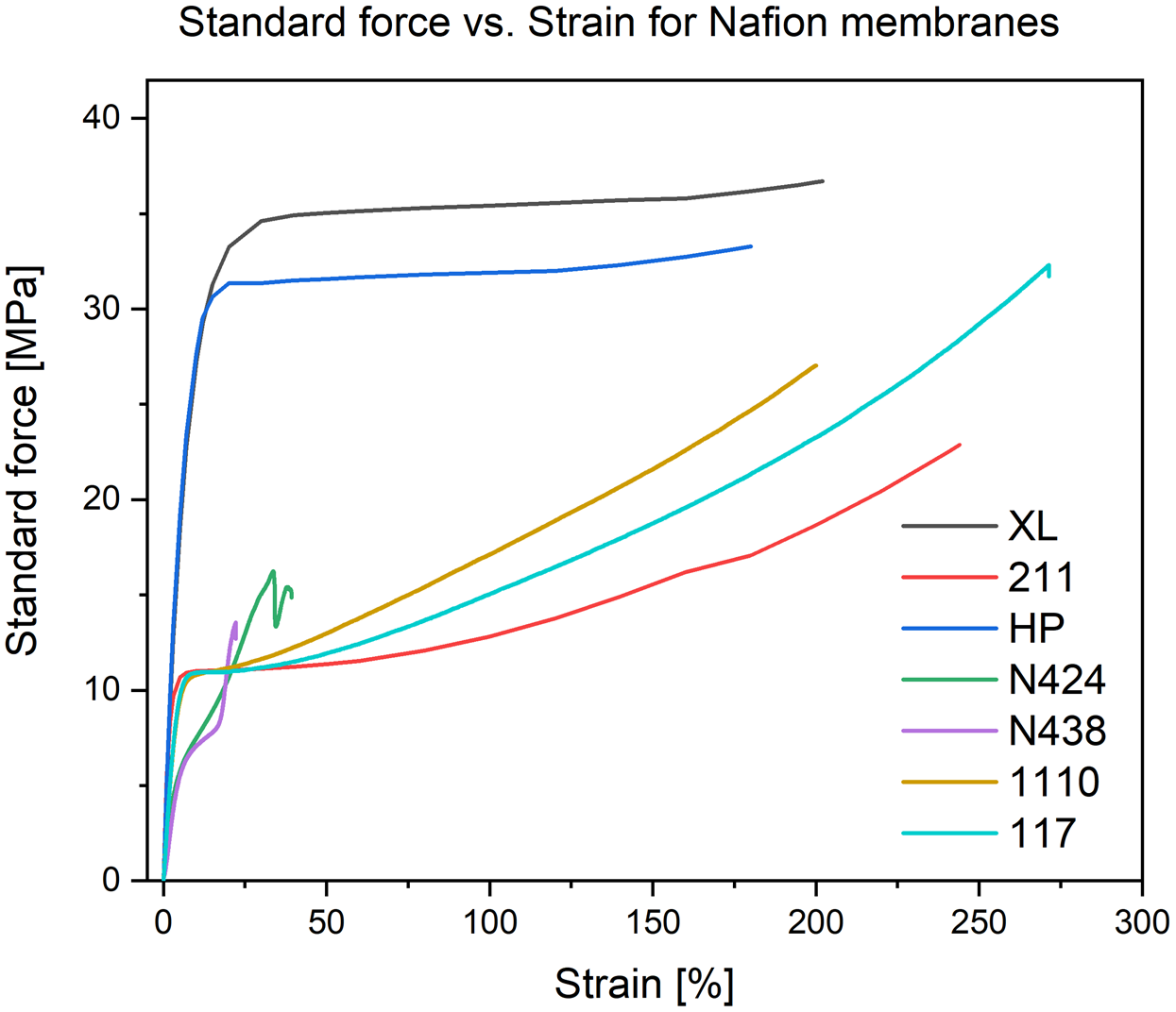
Tensile strength against strain of various Nafion PEM membranes.

**FIGURE 5:**
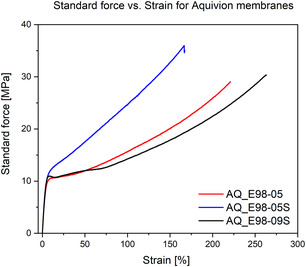
Tensile strength against strain of various Aquivion PEM membranes.

Results presented in Figures 4 and 5 show the standard force‐strain curves of the tested commercial membranes from Nafion and Aquivion. The membranes were tested up to the point at which the membrane either ruptures or deforms in such a way that no further force can be applied beyond the value reached (complete plastic deformation). We can notice that Nafion HP membrane can withstand the second highest applied force, but lacks the elasticity due to low strain percentage. Nafion XL achieved higher elasticity and higher forces [28]. Both membranes Nafion XL and HP have reinforced chemical structure to improve mechanical strength, which is clearly shown in comparison to other tested membranes. In contrast, membranes such as Nafion 117 and Aquivion E98‐09S showed exceptional elasticity, reaching strains higher than 250%. Nevertheless, those two membranes, along with many other membranes cannot withstand high forces and are therefore easily stretched.

An interesting phenomenon was observed with the reinforced membranes Nafion N424 and N438, where the membranes ruptured completely at strains below 50% and at forces below 20 MPa. To better understand this, we must point out that reinforced membranes are actually made from a net‐like structure of PTFE covered with a proton‐exchanging coating, whereas the rest of the membranes are more like solid polymer sheets. For this reason, the thin PTFE strains are easily deformed, which leads to the rupture of such membranes.

### 3.2 Thermal Degradation Study in Hydrochloric Acid

#### 3.2.1 SEM

**FIGURE 6:**
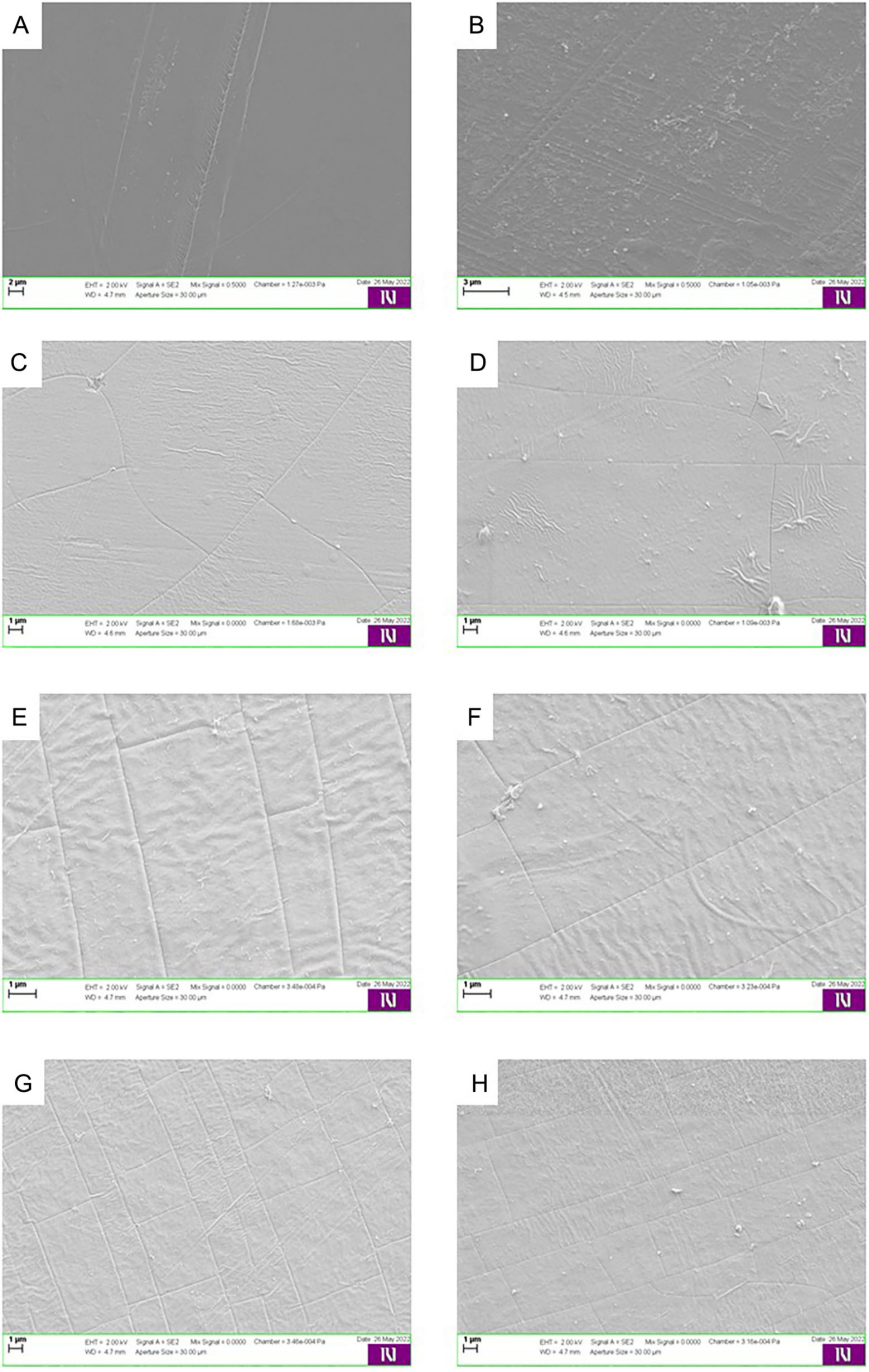
Nafion HP membrane, fresh (A) and used (B). Nafion 117 membrane, fresh (C) and used (D). Aquivion E98‐05S membrane, fresh (E) and used (F). Aquivion E98‐09S membrane, fresh (G) and used (H).

The fresh Nafion HP membrane had a smooth, intact surface with minimal defects (Figure 6A). After electrolysis, the SEM images showed increased roughness, scratches, and grooves, indicating chemical degradation due to prolonged exposure to acidic conditions and electrochemical stress (Figure 6B). The roughened surface suggests sulfonic acid leaching, which may reduce proton conductivity, increasing gas crossover, and affect long‐term mechanical stability. A similar observation was noted for the Nafion 211, Nafion N438, and Nafion N424.

In contrast to the latter membranes, the Nafion 117 membrane appeared relatively smooth, even after the batch electrolysis experiments, but exhibited increased roughness, wrinkles, and small cracks, indicating mechanical fatigue and chemical attack (Figure 6C,D). The membrane is clearly more suitable for HCl operation, but its long‐term stability may be questionable.

The fresh Aquivion E98‐05S membrane had a rougher surface than most Nafion membranes (Figure 6E). After electrolysis, the membrane exhibited a small increase in surface roughness and minor wrinkles but had fewer visible defects than Nafion membranes (Figure 6F). The lack of severe cracking indicates good chemical resistance, and makes Aquivion a potential alternative for prolonged electrolysis applications.

Similar observations were made for Aquivion E98‐09S, where only minimal structural changes were observed, as shown in Figure 6G,H.

Interestingly, membranes that demonstrated higher elasticity in tensile strength tests, such as Aquivion E98‐09S and Nafion 117, also showed comparatively less severe surface degradation under SEM. This suggests a possible correlation between mechanical flexibility and resistance to physical damage caused by electrochemical and thermal stress. More rigid or reinforced membranes, while structurally strong, may be more susceptible to crack formation and surface fatigue due to their limited capacity to accommodate mechanical strain during operation.

These results illustrate the influence of electrolysis conditions on membrane stability and emphasize the importance of selecting membranes with optimal chemical resistance. Future work should focus on incorporating stabilizing additives or alternative polymer structures to mitigate degradation and extend membrane performance in harsh electrochemical environments.

#### 3.2.2 FTIR‐ATR

The chemical stability of Nafion and Aquivion membranes subjected to hydrochloric acid electrolysis was investigated using FTIR‐ATR spectroscopy. The spectral analysis revealed distinct changes between fresh and used membranes in most cases, indicating degradation mechanisms affecting the polymer structure. To ensure reproducibility, each membrane sample was measured at multiple points on both sides. The resulting spectra showed minimal variation, confirming the consistency of observed chemical features.

**FIGURE 7:**
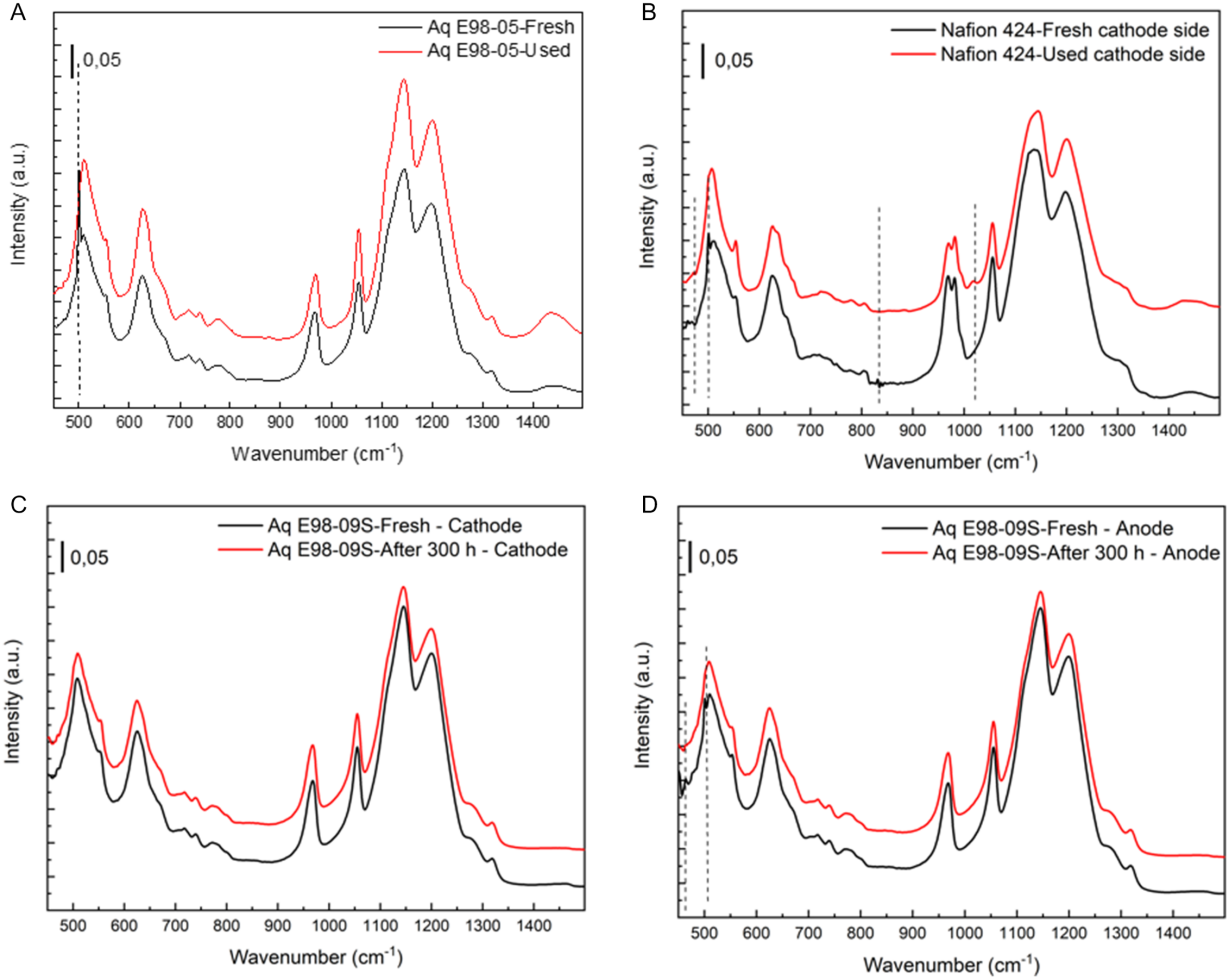
(A) FTIR‐ATR spectra of fresh and used Aquivion E98‐05 membrane; (B) FTIR‐ATR spectra of fresh and used cathode side of Nafion 424 membrane; (C) FTIR‐ATR analysis. Aquivion E98‐09S, cathode side; (D) FTIR‐ATR analysis. Aquivion E98‐09S, anode side.

The FTIR‐ATR spectra of both fresh and used Aquivion and Nafion membranes used in HCl electrolysis show a great similarity with the spectra reported in the literature, confirming the retention of the main structural features in the hydration process (Figure S1–S12). A major difference between the two membrane types lies in the region between 960 and 980 cm^−1^, where a characteristic doublet appears for the Nafion membranes, corresponding to the C–O–C stretching vibrations of the ether bonds in the main and side chains. Spectral changes near 501 cm^−1^ (Figure 7A) associated with the wagging vibration of the CF_2_ groups were observed in both membrane types, which could reflect a structural disorder or degradation of the fluorocarbon backbone [29].

Remarkably, a new band appeared at around 1020 cm^−1^ in the Nafion 424 membrane used, likely indicating interactions between chlorine species and the sulfonate groups, possibly forming chlorinated sulfonic acid derivatives [30] (Figure 7B). The spectral region around 800 cm^−1^ remains difficult to interpret as there is no clear assignment [31] changes in this region could result from Cl‐for‐F substitution or Cl–SO_3_ group interactions, both of which could contribute to chemical degradation. Importantly, the membranes with the most pronounced spectral differences were also mechanically fragile and brittle and often crumbled during handling, further supporting the presence of significant structural degradation.

These results highlight the impact of electrolysis conditions on membrane stability and emphasize the importance of selecting membranes with optimized chemical resistance. Among the membranes tested, the Aquivion samples showed slightly less pronounced spectral changes compared to their Nafion counterparts, indicating better chemical stability under the harsh conditions of HCl electrolysis. In particular, Aquivion E98‐09S exhibited a more consistent FTIR‐ATR profile with negligible deviations from the spectrum of the fresh membrane, indicating a lower extent of degradation. This relative resilience combined with better mechanical integrity after operation led to the selection of Aquivion E98‐09S for the subsequent long‐term electrolysis tests. Its performance in the initial trials emphasized its suitability for prolonged exposure to reactive chlorinated environments.

### 3.3 Electrochemical Degradation Study of Aquivion E98‐09S Membrane in HCl Medium

The chemical durability of the Aquivion E98‐09S membrane was evaluated after 300 h of continuous electrolysis in a MicroFlow cell operating under harsh conditions: 20 wt% HCl, constant current of 400 mA, and cell voltages reaching up to 2.8 V. The membrane was characterized using FTIR‐ATR spectroscopy before and after the experiment, with measurements taken separately on the anode and cathode sides. The resulting spectra are shown in Figure 7C,D.

The spectra of the fresh and used membrane (black and red lines, respectively) show no significant shift or disappearance of the characteristic vibrational bands. Key features include:

• ∼500–650 cm^−1^: bending modes of –SO_3_^−^ groups
• ∼1050–1150 cm^−1^: symmetric and asymmetric S=O stretching of sulfonic acid groups
• ∼1200 cm^−1^: CF_2_ backbone vibrations of the perfluorinated polymer matrix

The overall profile of the spectrum after 300 h remains virtually unchanged compared to the fresh sample. The spectral offset between the lines is intentional and does not indicate a change in intensity. Rather, it was introduced for better visual distinction. The lack of significant band loss or new features implies that the membrane's sulfonic acid functionalities remain intact, with no apparent chemical degradation on the cathode side under the applied conditions.

A similar observation is made for the anode‐facing side of the membrane. The postexperiment spectrum again mirrors that of the fresh membrane, with all major functional group peaks preserved. The sulfonic acid group bands and the CF_2_ backbone vibrations remain consistent in both shape and position. The visual offset in the spectra was applied solely for clarity.

These findings suggest that the Aquivion E98‐09S membrane exhibits strong chemical resilience in 20 wt% hydrochloric acid, even under prolonged electrochemical stress. The preserved vibrational features confirm the structural and chemical stability of the membrane's functional groups on both the anode and cathode sides. This indicates minimal, if any, degradation of the sulfonic groups after 300 h of operation, supporting its suitability for long‐term acid electrolysis applications.

## 4 Conclusion

This study demonstrates that while commercial PFSA membranes like Nafion provide robust mechanical properties, they are susceptible to chemical degradation when exposed to HCl/Cl_2_ environments, as evidenced by SEM and FTIR‐ATR analysis. In contrast, Aquivion membranes, particularly E98‐09S, maintained their chemical structure and performance during extended electrolysis trials. The Aquivion E98‐09S membrane showed the highest potential for long‐term stability in HCl electrolysis due to its minimal chemical changes and mechanical resilience.

These findings not only facilitate the selection of more suitable PEMs for halogen‐based applications but also highlight the need for further development of chemically stable, highly conductive, and mechanically robust membranes. Future efforts should explore composite structures and stabilizing additives to extend membrane lifetime in extreme electrochemical environments. In addition, the development and testing of non‐PFSA‐based membranes—which offer a more environmentally sustainable alternative—should be prioritized as such materials become more commercially accessible and capable of withstanding highly corrosive conditions like those present in HCl/Cl_2_ electrolysis systems.

## Supporting Information

Additional supporting information can be found online in the Supporting Information section.

## Author Contributions

**Alen Rupnik**: data curation (lead), formal analysis (lead), investigation (lead), methodology (equal), visualization (lead), writing – original draft (lead). **Janvit Teržan**: investigation (supporting), methodology (equal), validation (lead), writing – review and editing (equal). **Gleb Veryasov**: conceptualization (equal), supervision (equal). **Miha Grilc**: conceptualization (supporting), supervision (lead), writing – review and editing (supporting). **Blaž Likozar**: funding acquisition (lead), project administration (lead).

## Funding

This study was supported by Javna Agencija za Raziskovalno Dejavnost RS (P2‐0152); NextGenerationEU (HyBReED); H2020 Research Infrastructures (n°101058100).

## Conflicts of Interest

The authors declare no conflicts of interest.

## Supporting information

Supplementary Material

## Data Availability

The data that support the findings of this study are available from the corresponding author upon reasonable request.
