# Supplementary material for: (Electro)chemical and Mechanical Degradation Studies of Commercial Proton Exchange Membranes in HCl/Cl2 Systems for Hydrogen/Chlorine Production
Source: ChemistryOpen. 2026 May 10;15(5):e202500549. doi: 10.1002/open.202500549 (PMC13158368; doi:10.1002/open.202500549)
Supplement: Supplementary file 1 — Supplementary Material [file OPEN-15-e202500549-s001.pdf]

Supplementary Information

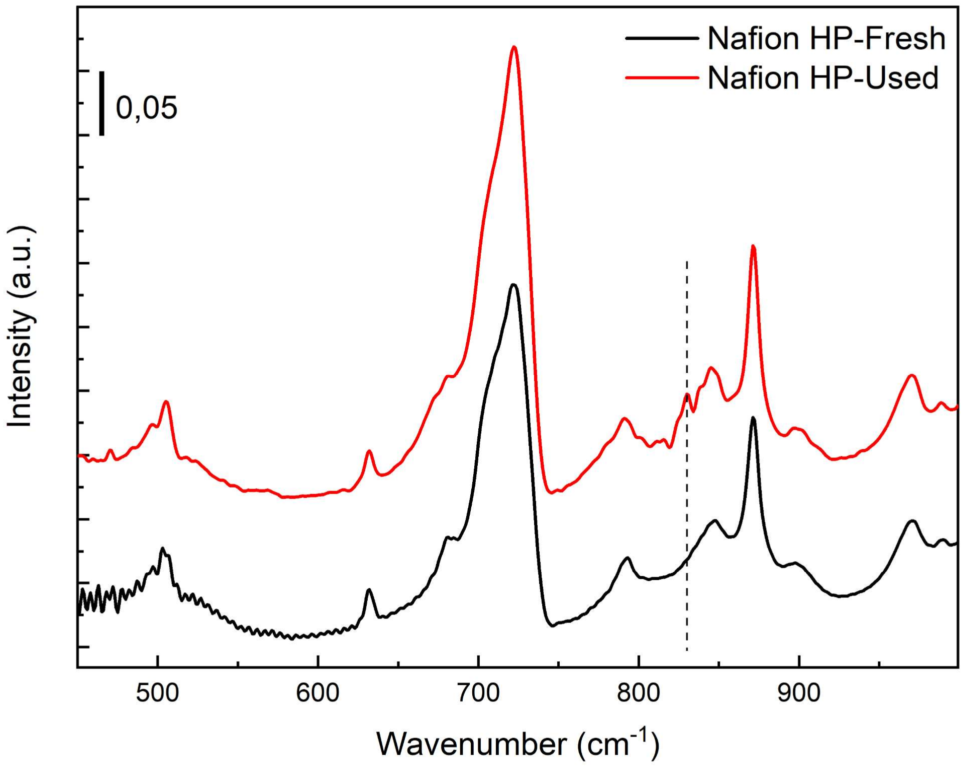

Figure S1. FTIR-ATR spectra of Nafion HP before and after HCl exposure.

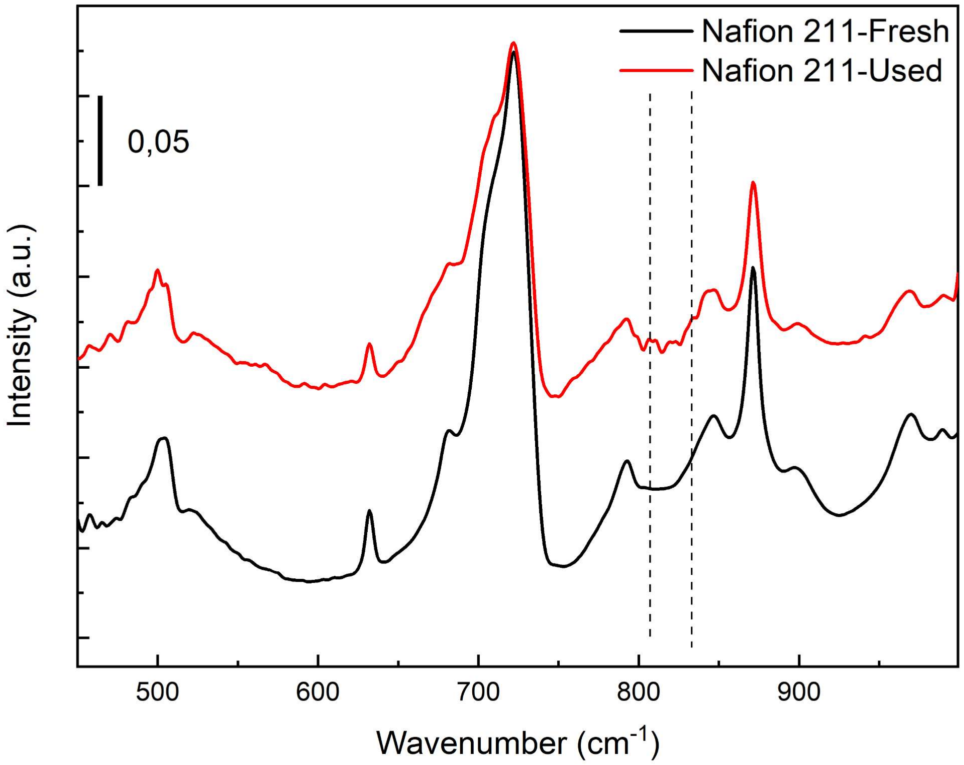

Figure S2. FTIR-ATR spectra of Nafion 211 before and after HCl exposure.

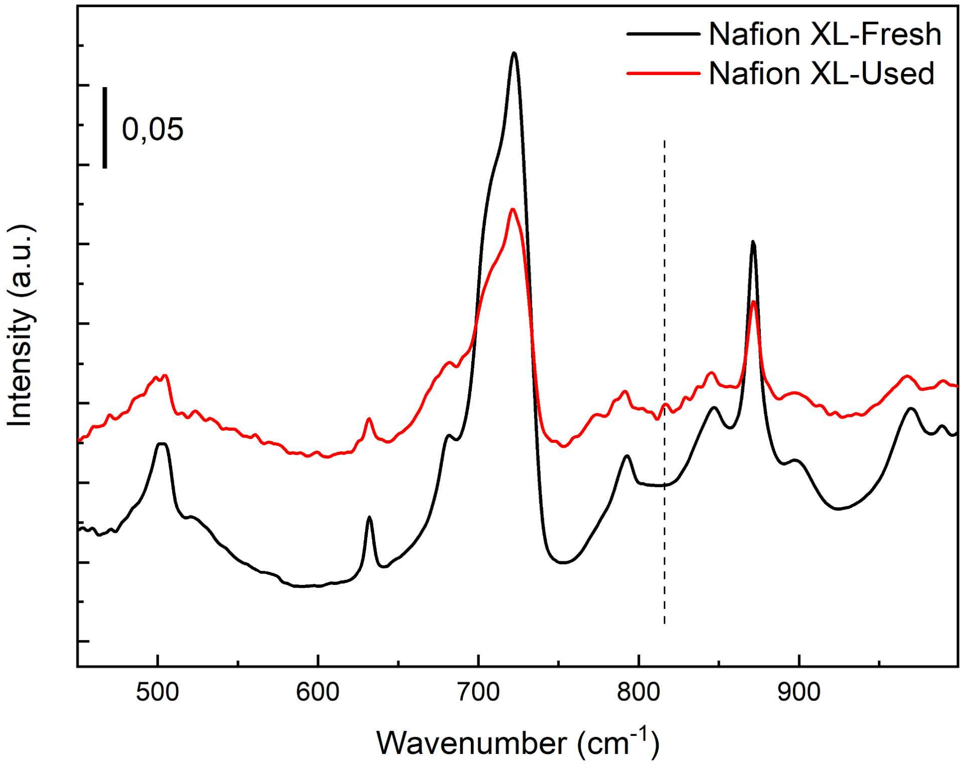

Figure S3. FTIR-ATR spectra of Nafion XL before and after HCl exposure.

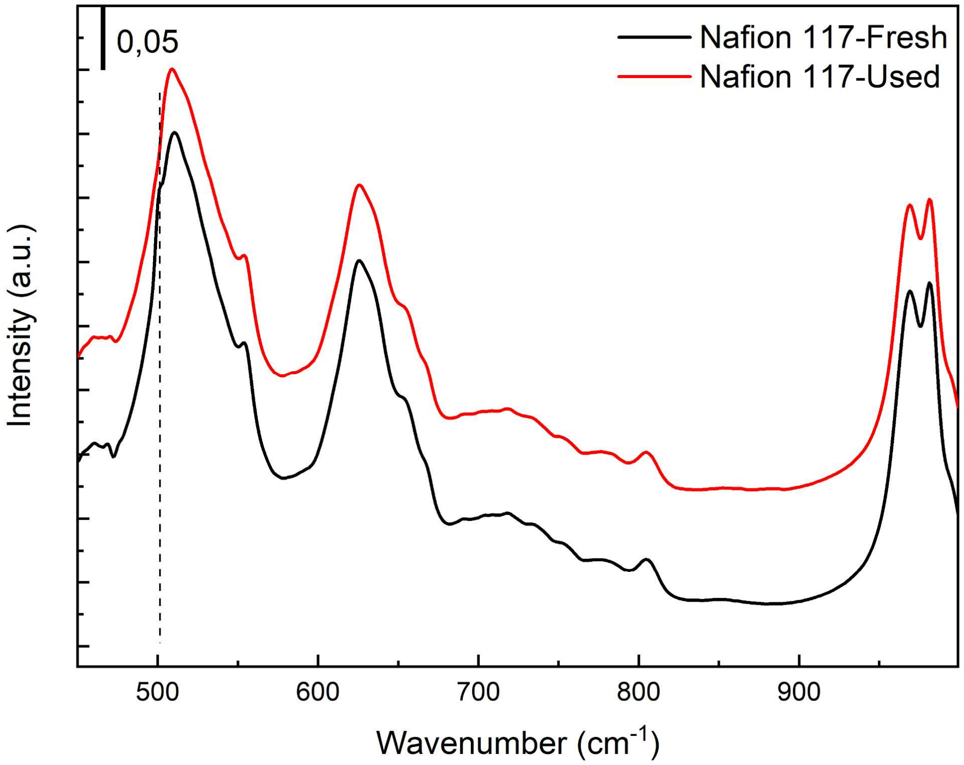

Figure S4. FTIR-ATR spectra of Nafion 117 before and after HCl exposure.

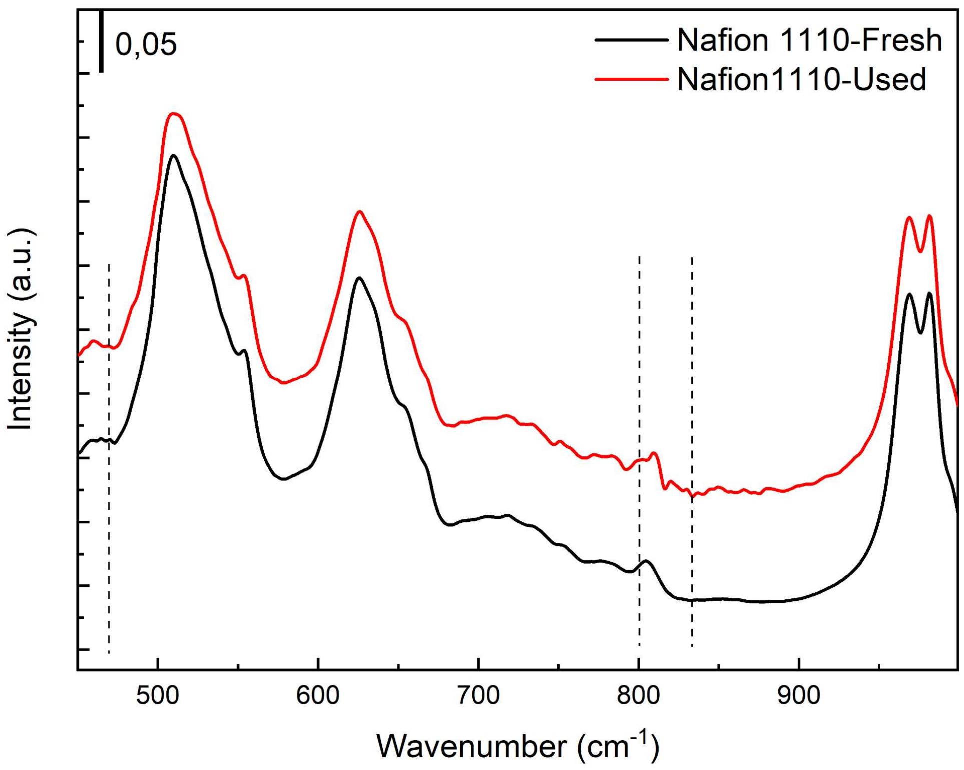

Figure S5. FTIR-ATR spectra of Nafion 1110 before and after HCl exposure.

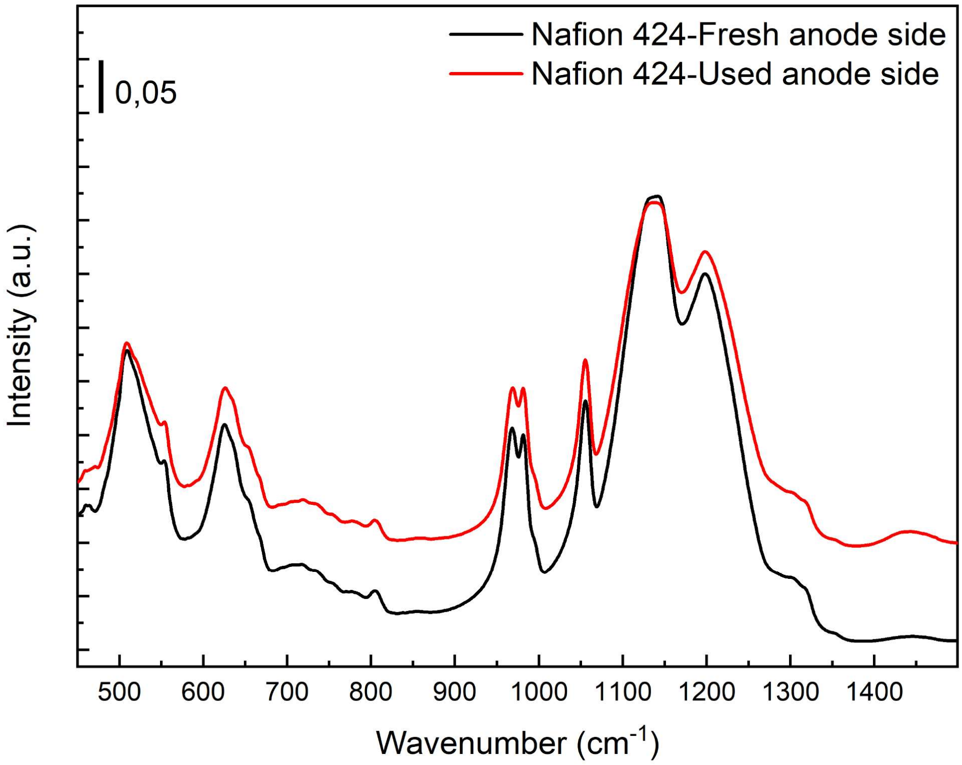

Figure S6. FTIR-ATR spectra of Nafion 424 (anode side) before and after HCl exposure.

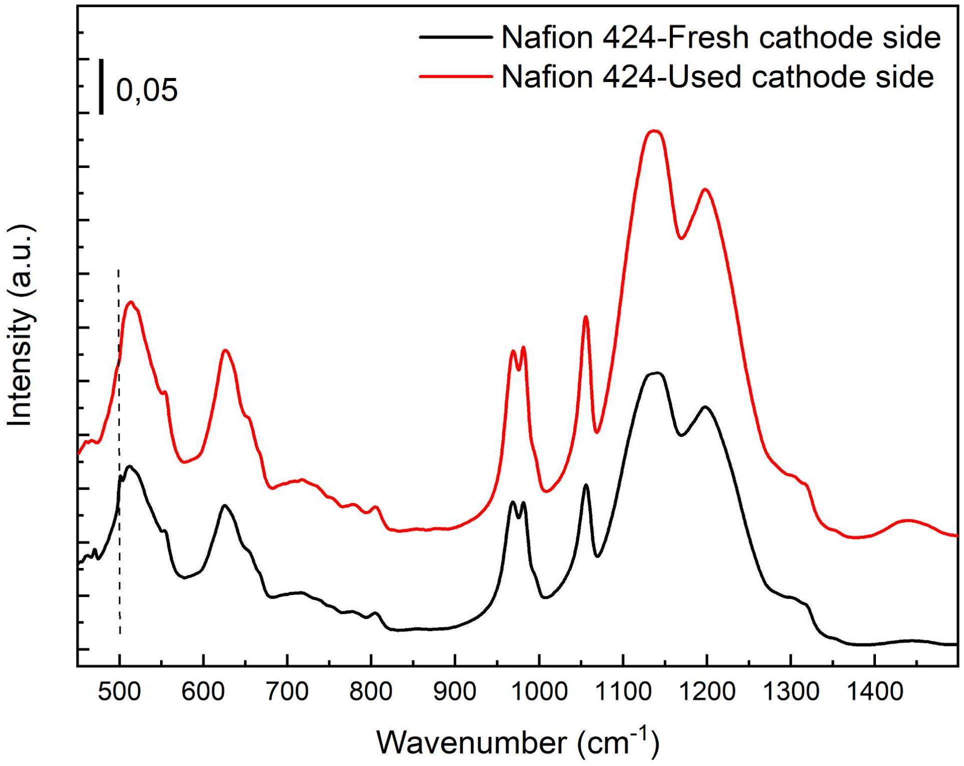

Figure S7. FTIR-ATR spectra of Nafion 424 (cathode side) before and after HCl exposure.

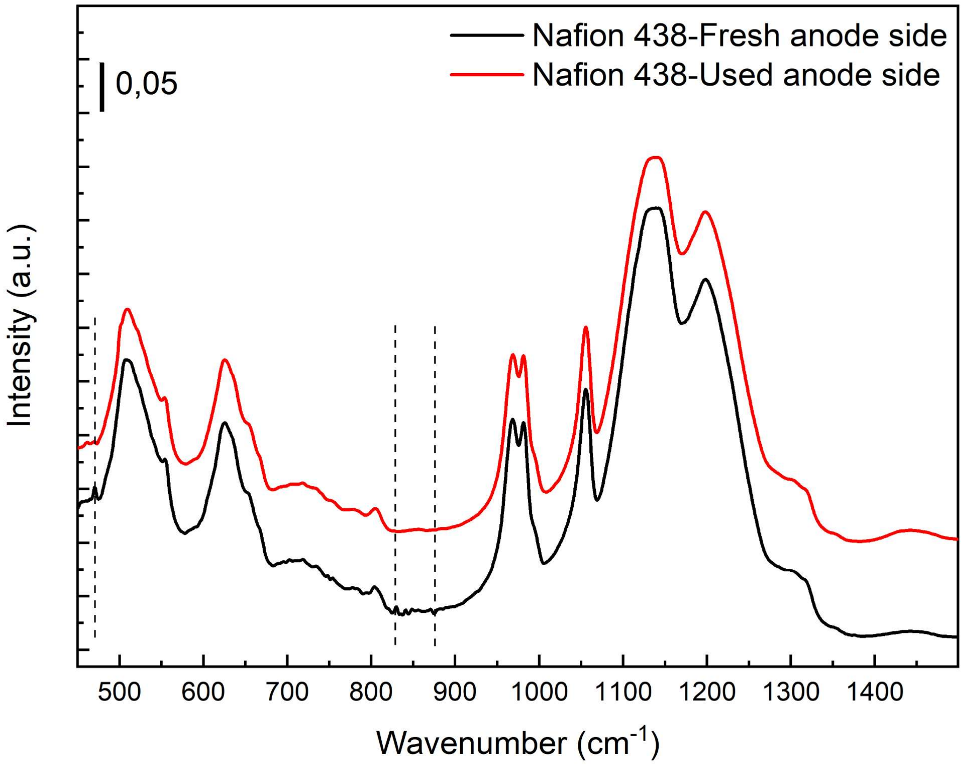

Figure S8. FTIR-ATR spectra of Nafion 438 (anode side) before and after HCl electrolysis.

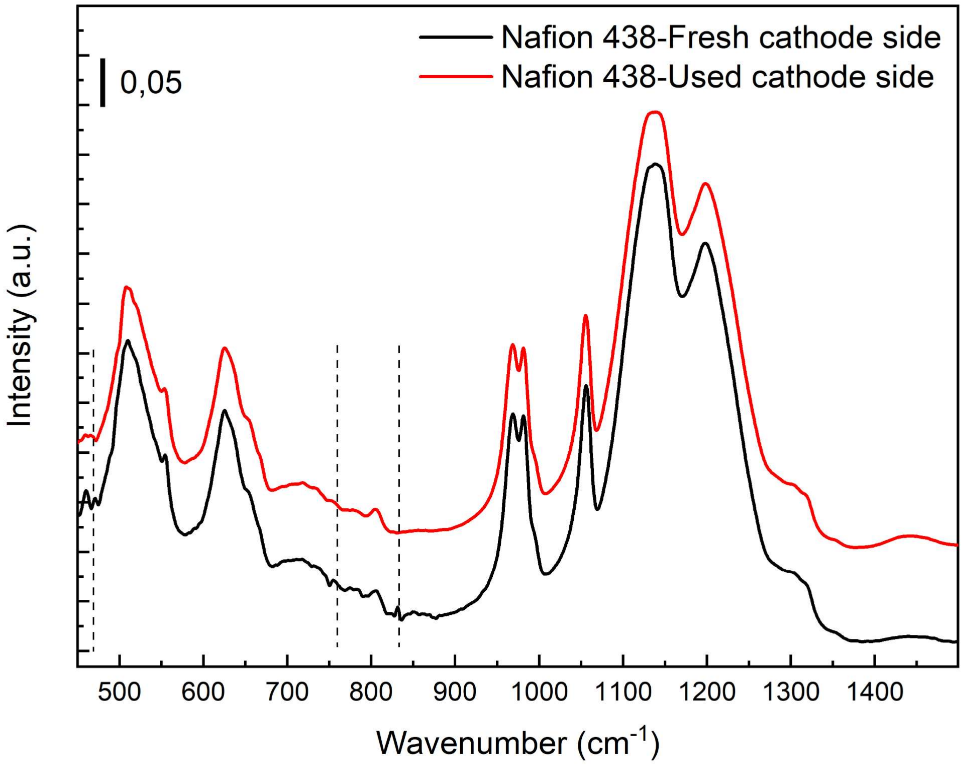

Figure S9. FTIR-ATR spectra of Nafion 438 (cathode side) before and after HCl exposure.

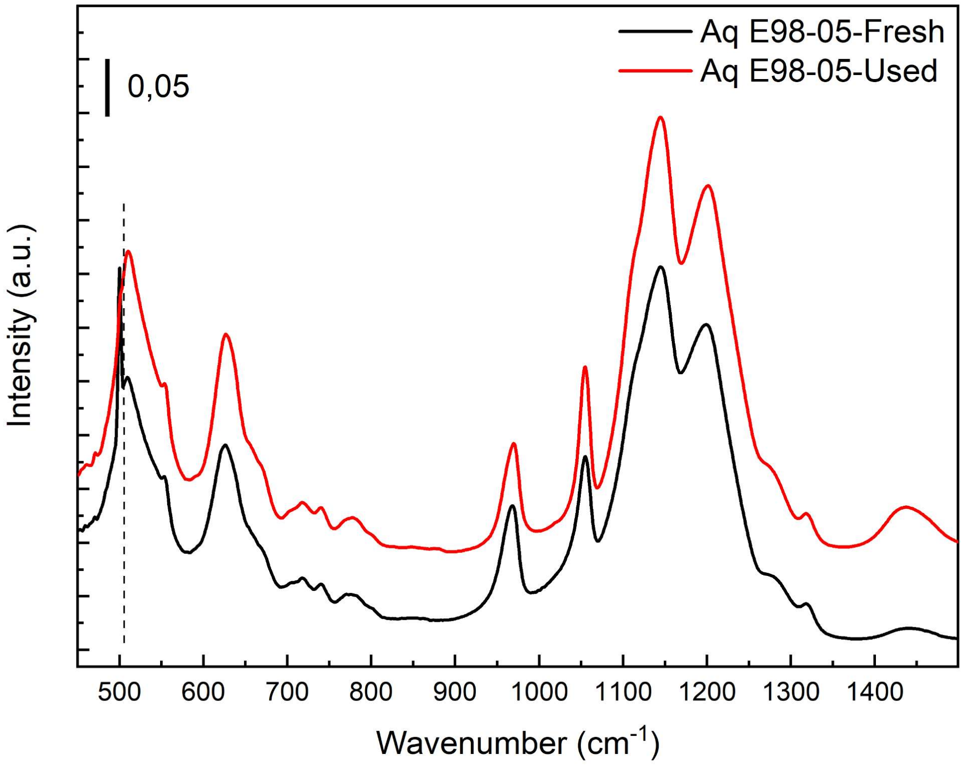

Figure S10. FTIR-ATR spectra of Aquivion® E98-05 before and after HCl exposure.

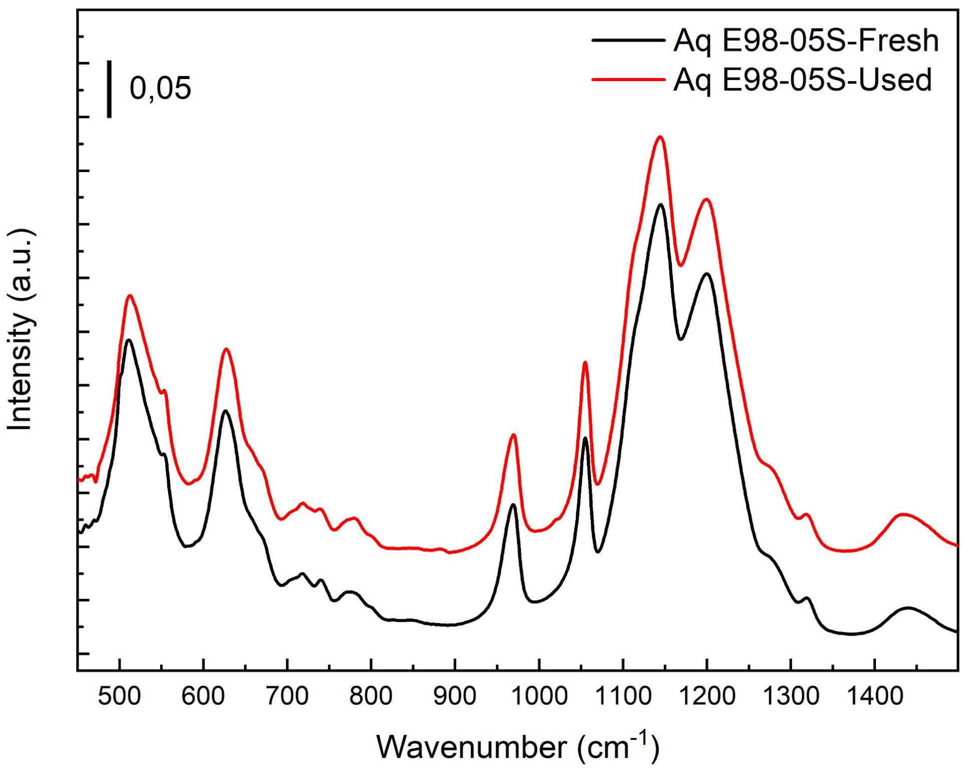

Figure S11. FTIR-ATR spectra of Aquivion® E98-05S. before and after HCl exposure.

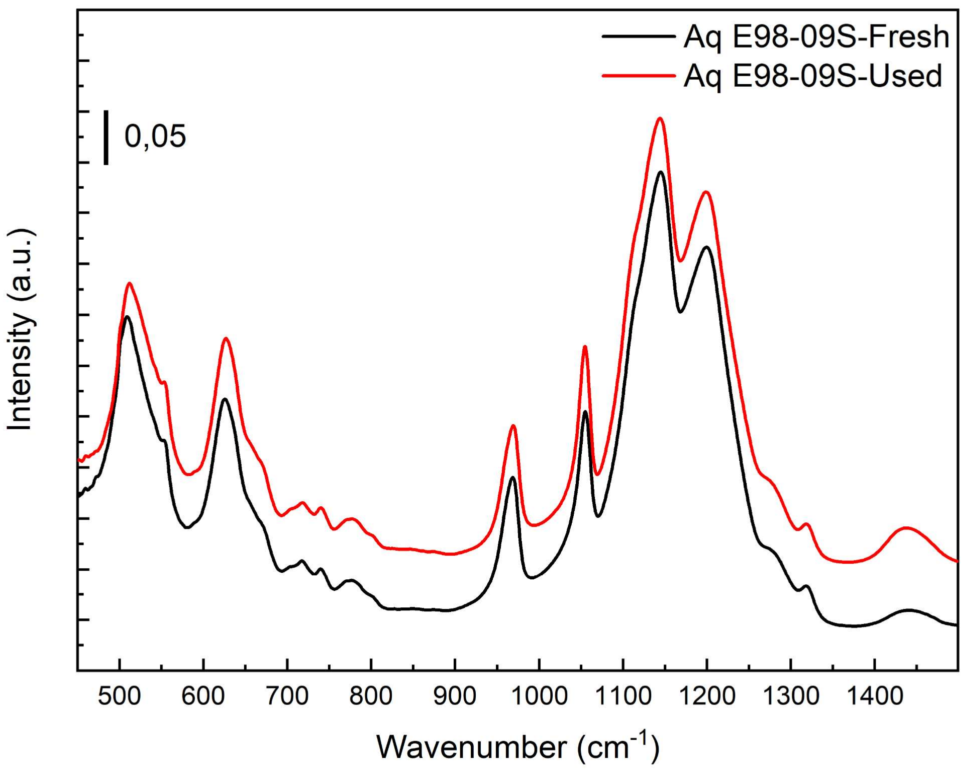

Figure S12. FTIR-ATR spectra of Aquivion® E98-09S before and after HCl exposure.
